# Targeting m6A Reader Phase Separation in Synaptic Plasticity: A Hypothesis-Generating Framework for Epitranscriptomic Drug Discovery

**DOI:** 10.3390/epigenomes10040064

**Published:** 2026-10-05

**Authors:** Reza Rastmanesh

**Affiliations:** Independent Researcher, Tehran 1939793694, Iran; r.rastmanesh@gmail.com

**Keywords:** m6A RNA methylation, YTHDF2, liquid–liquid phase separation, synaptic plasticity, epitranscriptomic drug discovery, FMRP, condensate pharmacology

## Abstract

Epitranscriptomic drug discovery has mostly targeted the catalytic “writers” (METTL3/METTL14) and “erasers” (FTO, ALKBH5) that deposit and remove N6-methyladenosine (m6A). That strategy just hit clinical validation: STC-15, a METTL3 inhibitor, has measurable anti-tumor activity from a first-in-human oncology trial, but the same structural features that made these enzymes attractive also limit them. Inhibition shifts m6A stoichiometry globally, while an m6A mark’s functional impact depends almost entirely on cellular context. YTHDF family reader proteins offer another angle. Their low-complexity domains drive liquid–liquid phase separation (LLPS). m6A binding stimulates this condensation across YTHDF paralogs—a property that matters for therapeutic selectivity. We propose a Phase Transition Threshold model in which YTHDF2’s condensation concentration decides whether synaptic mRNAs are degraded or maintained in translation-competent, FMRP-associated states. Recent evidence has established YTHDF2 synaptic localization, FMRP condensate formation in neurons, and functional antagonism between the two proteins. The central unresolved question is whether condensation in living cells is nucleation-driven rather than bulk-concentration-driven. If so, concentration-centered pharmacology misses the point. We present falsifiable predictions and a staged experimental program. A more fundamental risk is that YTHDF2-mediated decay may not require condensate formation at all. If that is the case, the therapeutic premise of this Perspective collapses.

## 1. The Orthodoxy and Its Limits

For the past decade, epitranscriptomic drug discovery has focused almost entirely on catalysis. Writers. Erasers. METTL3/METTL14 deposit m6A; FTO and ALKBH5 remove it. That strategy just paid off: STC-15, a METTL3 inhibitor, showed measurable anti-tumor activity in a first-in-human trial, but the same structural features that made these enzymes attractive—defined pockets—also constrain them. Inhibition alters m6A stoichiometry across the board. Yet an m6A mark’s functional effect depends almost entirely on where and when it appears.

STC-15 is a first-in-class METTL3 inhibitor from Storm Therapeutics. It triggers dsRNA accumulation and interferon signaling in preclinical models, remodels the tumor microenvironment, and sensitizes tumors to checkpoint blockade [1]. A Phase 1 dose-escalation trial (NCT05584111) saw tumor regressions across dose levels and tumor types—sustained partial responses included. Clinical activity correlated with interferon-signaling pathway upregulation [2,3]. STC-15 is the first RNA-methyltransferase inhibitor with proven human clinical activity. It provides real validation of the catalytic-inhibitor strategy—at least in oncology, where broad effects are acceptable.

But that success also reveals a limitation. STC-15 works through a global immunostimulatory effect—METTL3 loss, tissue-wide. The drug does not distinguish tumor from healthy tissue at the molecular level. That is acceptable in oncology precisely because indiscriminate effects are tolerated. Now think about transcript-specific effects downstream. In non-small cell lung cancer, METTL3 inhibition reduces chemoresistance by lowering m6A on ABCC2 mRNA and accelerating its decay—a YTHDF1-dependent effect [4]. But that specificity comes from a reader protein downstream, not from the drug itself. It is not hard-wired into the mechanism. It also does not demonstrate that YTHDF1-mediated decay requires LLPS—only that YTHDF1 is involved. Pharmacological METTL3 inhibition also gives lineage-selective effects in hematopoiesis [5]. This is encouraging: catalytic inhibition can have non-uniform consequences. But that selectivity comes from differential cellular vulnerability, not because the drug goes where it is needed.

In the brain, m6A function is sensitive to both dose and direction, but the evidence for each component of that claim comes from different studies. FTO expression fluctuates bidirectionally during memory formation: too much FTO activity impairs memory, and too little also impairs it [6]. Separately, knocking out Mettl14 in striatum disrupts synaptic function instead of improving learning [7]. These are not the same finding—one concerns eraser dosage, the other concerns writer deletion—and neither establishes that reader partitioning is the mechanism. The broader context-dependence of m6A function is reviewed in [8].

A temporally restricted catalytic inhibitor tells you when m6A is modulated but not where. It shifts m6A stoichiometry brain-wide—not within the specific circuit encoding a particular memory—and it cannot tell apart m6A sites that boost translation from those that trigger decay [8]. Is that a decisive objection? No. Is it a real limitation? Absolutely. To our knowledge, no one has directly compared the cognitive effects of reversible small-molecule METTL3/FTO inhibition against the constitutive genetic deletions above. They are not pharmacologically equivalent. It is possible that a well-timed, properly dosed catalytic inhibitor could be cognitively tolerable in ways that genetic ablation is not.

Our narrower claim: there is a complementary strategy. Target biophysics instead of chemistry. Develop it in parallel.

## 2. The Reader as a Structural Rheostat

### 2.1. Foundations: What Phase Separation Is, and Why the Evidence for It Needs Scrutiny, Not Just Citation

Rewind to 2009. That is when Brangwynne and colleagues watched *C. elegans* germline P granules moving like liquid droplets—dissolving and condensing in place rather than traveling anywhere [9]. That simple observation launched the LLPS field.

Biomolecular condensates are membraneless compartments that concentrate proteins and nucleic acids. They organize RNA metabolism, DNA damage responses, and signal transduction [10]. The basic principle? Multivalent, weak, reversible interactions among intrinsically disordered regions. Later, researchers formalized the biophysics of dilute-and-dense-phase coexistence [11]. For prion-like RNA-binding domains, they worked out a “molecular grammar”—cation-π and π-π interactions that govern phase behavior [12].

So where does the m6A reader field’s central biophysical claim fit? Right here: YTHDF proteins undergo LLPS through their LCDs. YTH-domain readers bind m6A via a conserved aromatic cage—YTHDC1’s structure shows it [13]. The fold is conserved across YTHDF paralogs, and YTHDF1, YTHDF2, and YTHDF3 all have N-terminal glutamine-rich low-complexity domains that drive LLPS [14,15].

Add m6A to the mix and phase-separation propensity goes up. m6A acts as a multivalent scaffold for reader condensation [14]. You see this in several non-neuronal disease contexts. Chronic liver disease models show enhanced YTHDF2 phase separation with m6A-modified mRNA binding [16]. Arsenite-induced oxidative stress triggers m6A-dependent YTHDF2 phase separation that promotes malignant keratinocyte transformation by suppressing PTEN translation [17,18].

Then there are the reconstitution experiments. Recombinant YTHDF2 forms 1,6-hexanediol-sensitive liquid droplets in vitro via a conserved glutamine-rich LCD segment. m6A-modified RNA oligonucleotides stimulate droplet formation—strongly. A YTH-domain point mutant that cannot bind m6A? Fails to phase-separate in vitro and in cells. Here is the kicker: this is not just YTHDF2. Other YTH-domain m6A readers phase-separate through the same mechanism [19]. The selectivity implications are immediate.

Cross-context replication? We see it as meaningful corroboration. LCD-driven, m6A-stimulated condensation really is a YTHDF family property. But reader-specificity? Not so much. Not in any way that would help with selective drug targeting. And definitely not evidence for the synapse.

Every study here is from non-neuronal systems—and that is a problem. Since the compositional basis for phase separation is shared across YTHDF1–3, the underlying biophysics is family-wide, so any drug-discovery program built on this mechanism inherits a selectivity problem. This problem is discussed further below.

Now, a caveat before we get carried away. The field’s go-to methodological reference catalogs what can go wrong with in vitro LLPS assays: non-physiological salt; crowding agent concentrations that do not match cells; overexpression artifacts that distort what is actually happening; and 1,6-hexanediol is not a perfect test for liquid-like character [20]. Several findings above came from exactly these conditions: reconstitution experiments with purified LCD fragments at micromolar concentrations in high concentrations of polyethylene glycol (PEG) and non-physiological buffers. That does not invalidate the findings. But we cannot assume they translate to an intact, crowded, ion-buffered neuronal cytoplasm. Cross-context replication in equally artificial in vitro systems is not physiological relevance.

One further concern deserves explicit statement. Most of the cellular evidence for YTHDF2 condensate formation comes from overexpression systems. Whether endogenous YTHDF2—expressed at physiological levels, in a neuronal cytoplasm with its full complement of competing RNA-binding proteins—forms condensates of the kind observed in reconstitution or overexpression studies has been a legitimate question. Recent work has begun to address this. Zou et al. [21] showed that YTHDF1 and YTHDF2 differ in their low-complexity domains and exhibit different behaviors in condensate formation and subsequent physiological functions. This suggests that endogenous condensate formation is being studied, even if the evidence remains incomplete.

The molecular grammar framework [12] predicts that cation-π interactions between arginine residues in the YTHDF2 LCD and aromatic residues in partner proteins, together with π-π stacking among aromatic residues, are the dominant driving forces for condensation. This framework was derived from prion-like RNA-binding domains, and its applicability to YTHDF2 specifically is an extrapolation. Mutating these residues should reduce condensate formation without affecting m6A binding—a prediction that is testable with the YTHDF2 LCD mutants described in Tier 2.

### 2.2. Mechanism of Reader-Driven mRNA Fate

YTHDF2 binds an m6A-marked mRNA and recruits the CCR4-NOT deadenylase complex, leading to transcript decay [22]. This recruitment has been demonstrated to occur through direct protein–protein interactions. The additional hypothesis—that LCD-driven condensate assembly is required for this recruitment—remains to be directly demonstrated. The condensate may be a facilitator, a consequence, or an epiphenomenon. The model treats condensate formation as the mechanism, but the mechanism may be the recruitment itself, with condensation as a downstream architectural feature.

This distinction matters for pharmacology. If condensate formation is required, then shifting C-sat or nucleation kinetics is a viable drug strategy. If condensate formation is not required—if YTHDF2-mediated decay proceeds through direct CCR4-NOT recruitment regardless of condensate status—then the therapeutic premise of this Perspective collapses. We flag this in Section 3 as a failure criterion. It is the model’s second-most-serious vulnerability, after the nucleation-versus-concentration question.

### 2.3. A Working Model, Presented Alongside Its Competitors

What about the synapse? YTHDF2 interacts with Fragile X Mental Retardation Protein (FMRP) there. FMRP represses translation. It binds some m6A-modified transcripts and stalls ribosome elongation [23,24].

FMRP’s role in neuronal translational control has been well established through non-condensate mechanisms. The foundational CLIP study that defined FMRP’s mRNA targets showed this: FMRP is a polyribosome-associated RNA-binding protein that stalls ribosomal translocation on synaptic transcripts and autism-risk genes [23]. It is the standard any new model has to measure up to.

Setting the m6A literature aside for a moment—FMRP itself can phase-separate. Purified FMRP does it in vitro with RNA, forming liquid droplets through its C-terminal low-complexity disordered region. It is phosphoregulated, which fits with activity-dependent translational control. During transport, mRNAs sit in a repressed, condensed state. Synaptic stimulation and FMRP dephosphorylation release them [25]. This finding is necessary for the model we are proposing. Without it, we would have no biophysical basis for a condensate-partitioning account of FMRP/YTHDF2 competition. But necessary does not mean sufficient. The authors of that study never actually show that the phase-separated state is required for translational repression in an intact cell. It could be correlated [25,26].

Two prerequisites for the synaptic model deserve explicit statement. First, YTHDF2 must be present in dendrites and at synapses. Recent evidence has established this. Flamand and Meyer [27] identified YTHDF2 and YTHDF3 as mediators of m6A-dependent mRNA localization to neurites, using single-molecule FISH in hippocampal neurons. Niu et al. [28] showed that YTHDF2 is highly expressed in mouse retinal ganglion cells and that conditional knockout leads to increased dendrite branching and more synapses in the inner plexiform layer. And Koo et al. [29] demonstrated that YTHDF2 interacts with motor proteins, translational regulators, and microtubules to facilitate distal transport of m6A-tagged mRNA in developing neurons. The presence of YTHDF2 in neuronal processes is no longer a prerequisite uncertainty—it is established.

Second, FMRP must form condensates in neurons. The in vitro evidence [25] is strong, and recent work has begun to establish this in vivo. The Koo et al. [29] study demonstrates that FMRP interacts with the YTHDF2 transport complex in neurons. A 2026 review on FMRP in neurodegenerative diseases describes FMRP forming ribosome-rich condensates in axons and dendrites. The formation of FMRP condensates in neurons is now supported by multiple lines of evidence, though the precise composition and dynamics of these condensates remain to be characterized.

One possibility: mutually exclusive condensate partitioning. FMRP binds the mRNA body. YTHDF2 grabs the m6A mark. Competition—when it happens—is about which compartment a transcript ends up in. The general model, in which YTHDF2 concentration relative to its saturation concentration (C-sat) determines whether synaptic transcripts are translated or degraded, is shown schematically in Figure 1. A YTHDF2 condensate that kicks out ribosomes and promotes decay? Or an FMRP condensate that keeps stalled ribosomes and supports translation?

There are four alternatives, including our proposal:

1. **Mutually exclusive condensate partitioning (our proposal).** Transcripts sort into non-overlapping YTHDF2 or FMRP condensates.
2. **Co-occupancy.** YTHDF2 and FMRP bind the same transcripts together. Downstream cofactor recruitment determines fate, not compartment identity.
3. **Kinetic competition.** Partitioning comes down to binding and unbinding rates, not which compartment.
4. **Network-mediated regulation.** FMRP modulates translation of lots of other condensate-forming synaptic proteins [30]—postsynaptic density components, active zone proteins, synaptic vesicle condensates. This shapes transcript fate indirectly through the broader condensate network, not via direct YTHDF2 competition.

The network-mediated model deserves more development than it has received. Under model 4, FMRP does not compete with YTHDF2 directly. Instead, it regulates the expression and localization of the broader set of condensate-forming proteins that shape the synaptic RNA environment. Transcript fate is determined not by which of two condensates a transcript enters, but by the composition and dynamics of the condensate network as a whole. FMRP loss would alter the network, and YTHDF2’s partitioning would shift as a downstream consequence—without any direct YTHDF2/FMRP interaction. The recent Koo et al. [29] paper, however, provides evidence that bears directly on the competition model. Koo et al. [29] reported that FMRP acts as a context-guiding interactor that reshapes the YTHDF2 complex by recruiting specific cofactors and motor proteins, thereby promoting transport rather than degradation of m6A-tagged transcripts. This is direct evidence for a competition-like mechanism. FMRP and YTHDF2 are not merely coexisting in the same cellular space—they are functionally antagonistic with respect to transcript fate. The mechanism appears to involve FMRP reshaping the YTHDF2 complex by recruiting specific cofactors and motor proteins, thereby promoting transport rather than degradation of m6A-tagged transcripts.

This finding significantly strengthens the competition model. It does not, however, resolve which of the four competition models is correct. The evidence is consistent with model 1 (mutually exclusive partitioning), model 2 (co-occupancy with competitive cofactor recruitment), and model 3 (kinetic competition). Distinguishing among these remains an open experimental question.

Model 1 generates the sharpest, most immediately falsifiable predictions: direct co-localization or its absence; and direct co-occupancy or its absence. The required methods—imaging and reconstitution—already exist. That makes model 1 the most tractable entry point, not necessarily the best-supported mechanism (Figure 2, panel A). But we now propose that model 4 be tested in parallel, using network perturbation approaches described in Tier 1b. If the Tier 1 experiments return a negative result—FMRP and YTHDF2 consistently co-localizing rather than segregating—that result would favor models 2–4. It should redirect the field toward whichever of those the data support.

Three predictions distinguish model 1 from its alternatives:

Take in vitro reconstitution first. Mix purified YTHDF2 LCD, FMRP LCD, and model mRNAs. You should see two distinct condensate populations with mRNAs partitioned between them.

Then there is super-resolution microscopy. In neurons, it should reveal spatially separate YTHDF2 and FMRP condensates at synapses.

Finally, co-immunoprecipitation. Under phase-separating conditions, YTHDF2 and FMRP should rarely show up on the same mRNA.

A stronger test: does co-occupancy increase specifically when phase separation is pharmacologically or genetically blocked? That result would favor model 2 or 3.

We should be clear about a limitation of the co-IP prediction. Co-immunoprecipitation of RNA-binding proteins on the same mRNA requires crosslinking and immunoprecipitation under conditions that preserve condensate integrity. If condensates are disrupted during lysis—and many standard lysis buffers do disrupt them—the co-IP result will reflect post-lysis binding, not in vivo partitioning. The prediction is testable in principle, but only with lysis conditions validated to preserve condensates and with appropriate controls (e.g., a non-condensate-forming mutant). Without those controls, a negative co-IP result is uninterpretable.

Selectivity within the YTHDF family remains a substantial open problem. We flagged this in Section 2.1 as a family-level rather than reader-specific property. The problem persists independent of which competition model proves correct. A compound engaging the YTHDF2 LCD will likely engage YTHDF1 and YTHDF3 as well. Their compositional basis for LLPS is shared. Chimeric LCD constructs and alanine-scanning mutagenesis might identify YTHDF2-selective hotspot residues in principle. But can we achieve functional selectivity at all in a system built on multivalent, low-affinity interactions? Modest perturbations can ripple across several condensate systems simultaneously. Binding-affinity data alone cannot answer this question.

Figure 2 compares the four models side by side. The comparison is intended to clarify which predictions are shared and which are model-specific.

## 3. The Thermodynamics of Plasticity and Psychopathology

### 3.1. What C-Sat Is, How It Would Actually Be Measured, and Why That Measurement May Not Be the Right Target

Disease relevance? We are framing it around a Phase Transition Threshold. The key parameter is C-sat—saturation concentration—the point where a protein–RNA mixture splits into two phases. Figure 1 shows the model schematically, with the diffuse and condensed regimes separated by C-sat. In cells, however, true C-sat is not directly measurable. We therefore distinguish thermodynamic C-sat from an observed condensation threshold (C-obs). C-obs is the expression level at which condensate number begins to increase sharply. It is an inferred proxy, not a thermodynamic saturation concentration, and it depends on nucleation, observation time, and local concentration. Do not confuse this with condensate dynamics. They are different. Fluorescence recovery after photobleaching (FRAP) and fluorescence correlation spectroscopy (FCS) measure diffusion and exchange kinetics inside a condensate that is already there. They tell you how liquid-like a droplet is. Not what concentration made it form.

To measure C-sat, you need titration. Typically: image the dilute-phase protein concentration across a series of concentrations. Find the plateau where adding more protein just goes into the dense phase instead of raising the dilute-phase concentration. We are spelling this out because the whole pharmacological logic hangs on C-sat being measurable. Get the measurement wrong and the experiments in Section 5 do not mean much.

But measuring C-sat in a neuron is not the same as measuring it in a cuvette. In a test tube, you control the total protein concentration and let the system equilibrate. In a neuron, you cannot set the concentration—you can only manipulate expression and observe the result. The dilute-phase concentration in a subcellular compartment is not directly observable with current methods, though FCS can provide estimates. What you can measure is condensate number, size, and intensity, from which C-sat must be inferred under assumptions about local volume and diffusion that may not hold. Local crowding, active transport, and competing RNA partners all shift the effective concentration in ways that bulk measurements miss. The Tier 2 experiments described in Section 5 will attempt this measurement, but we should be honest that no precedent exists for C-sat measurement of an endogenous RNA-binding protein in intact neurons. If the measurement proves infeasible, the C-sat-centered pharmacological program is not testable in its current form, and the framework would need to pivot toward nucleation kinetics or network-level perturbations.

But there is a bigger question before we even get to measurement. We think this is the framework’s single most serious vulnerability: Does bulk concentration actually control YTHDF2 condensation in a living neuron?

In a reconstituted in vitro system, concentration is just a number. Well-mixed. Phase separation above C-sat happens passively—you cross the threshold, it demixes. In a cell? Not so simple. Local concentration is not uniform. Crowding shapes it. So do competing partners, transport, and compartment geometry. And in vivo, condensates often form through nucleation—seeded by a scaffold, an RNA structure, or a localized trigger. Not passively. Not just because you crossed some bulk threshold. The two scenarios—concentration-driven and nucleation-driven—are shown side by side in Figure 1, and they lead to fundamentally different pharmacological strategies.

If synaptic YTHDF2 condensation is nucleation-driven rather than bulk-driven, then a drug that “raises C-sat” is going after the wrong thing. The real lever might be nucleation kinetics—say, the availability of a scaffolding RNA or protein. That is a different medicinal chemistry problem. Harder in some ways than shifting a thermodynamic threshold.

What would a nucleation-driven model look like? Under this account, a specific RNA structure, a locally enriched scaffold protein, or a post-translational modification event seeds condensate formation at a defined subcellular site. The concentration of YTHDF2 in the compartment is permissive but not determinative: condensates form where the seed appears, not where the concentration crosses a threshold. The pharmacological target becomes the seed, not the concentration. This changes everything about the screening strategy. Instead of looking for compounds that shift C-sat, you look for compounds that block or promote seed formation. Whether such compounds exist for YTHDF2 is entirely unknown. We do not have data distinguishing nucleation-driven from concentration-driven condensation for YTHDF2 in any cell type. It is a prerequisite uncertainty. Tier 1–2 experiments in Section 5 have to sort it out. Only then can a C-sat-centered program be considered well-targeted rather than merely plausible.

### 3.2. Disease-Relevance of m6A: Strong; Disease-Relevance of Reader Partitioning Specifically: Unestablished

Substantial empirical support exists for the premise that m6A dysregulation contributes to synaptic and cognitive dysfunction. Almost entirely through a writer/eraser lens, not a reader-partitioning one. The inferential gap between these two literatures is substantial and worth stating explicitly: evidence that writer or eraser manipulation alters cognition does not establish that reader partitioning is the mechanism. It establishes that m6A stoichiometry matters. Whether the downstream effect operates through reader condensates, reader binding without condensation, or non-reader pathways remains untested.

A tiered confidence assessment of the model’s constituent claims is provided in Table 1.

Consider the Alzheimer’s evidence. A conserved reduction in m6A modification—associated with aging and neurodegeneration—correlates with impaired synaptic plasticity and reduced translation of Camk2a and Glua1 transcripts. This holds in both Alzheimer’s disease models and human cingulate cortex [31]. METTL3-mediated m6A modification enhances long-term memory consolidation in the mouse hippocampus [32]. Reduced METTL3 expression has been reported in Alzheimer’s disease brains, where it correlates with synaptic loss and cognitive impairment [33]. METTL16-mediated methylation of MAT2A mRNA improves hippocampal synaptic plasticity, dendritic spine morphology, and learning/memory in a 5× FAD mouse model [34,35]. Beyond Alzheimer’s, a broader psychiatric epitranscriptomics literature links aberrant m6A regulation to depression, anxiety, schizophrenia, and bipolar disorder [36].

This evidence reinforces the premise that m6A dysregulation matters for cognitive disease. What it does not test: whether reader-condensate partitioning—rather than deposition/removal balance—is the operative mechanism. The Alzheimer’s findings are also correlational. m6A reduction may be a consequence of synaptic dysfunction rather than a cause. The direction of causality has not been established in either the mouse models or the human tissue.

Hyper-condensation (lowered C-sat). Rett syndrome is a neurodevelopmental disorder marked by cognitive rigidity. Whether it involves m6A dysregulation specifically has not been established in the literature we could verify. We do not cite a source for that claim. Our hypothesis—presented as a conjecture, not a claim—is that C-sat is pathologically lowered in this context. YTHDF2 condensates could form spontaneously, or fail to disassemble after a plasticity stimulus has passed. They may transition from dynamic liquid droplets toward persistent, gel-like states—a transition documented for other LCD-containing proteins [37]. But there is no direct evidence linking Rett syndrome to YTHDF2 hyper-condensation, and no YTHDF2 condensate measurements have been reported in Rett models. The hypothesis rests entirely on the general role of m6A in synaptic scaling. It requires C-obs measurement in Rett-model neurons (Tier 2) before it can be evaluated.

Hypo-condensation (elevated C-sat). Addiction might be the opposite case. We hypothesize—without direct evidence—that transcript surges in the nucleus accumbens during drug exposure could overwhelm YTHDF2’s clearance capacity, exploiting the finite client-molecule capacity of phase-separated condensates [38]. Direct epitranscriptomic evidence for this hypothesis is thin. A single postmortem pilot study of human nucleus accumbens tissue in alcohol use disorder drew from three subjects per group. It found differential m6A methylation in 26 hypermethylated and three hypomethylated mRNAs, with most differentially methylated transcripts enriched for immune-related pathways, not synaptic-plasticity genes [39]. A study of this size cannot support a directional claim about YTHDF2 condensation in addiction. We therefore present the addiction model as an untested conjecture with no empirical support. The broader nucleus accumbens epigenetics literature documents durable, transcript-specific chromatin and transcriptional remodeling following chronic cocaine exposure [40]. But this concerns histone and chromatin modification—a mechanistically distinct system. It should not be read as direct support for the m6A/reader hypothesis.

We propose a direct discriminating experiment. Titrate transcript load in vitro at fixed YTHDF2 concentration. The finite-capacity model predicts a sharp threshold effect. Competing explanations predict something else: a graded decline. Reduced YTHDF2 expression. Altered post-translational modification. Declining m6A deposition. A complementary test: is clearance failure global? Or transcript-specific and abundance-sparing? Global failure would be consistent with bulk condensate saturation. Transcript-specific failure would be consistent with per-transcript binding kinetics.

**Key falsifiable predictions and failure criteria**

We would regard the model as challenged or falsified by any of the following: YTHDF2 and FMRP consistently co-localizing within the same condensates under normal physiological conditions; manipulation of YTHDF2 phase behavior having no effect on neuronal mRNA decay kinetics; increasing transcript load failing to produce a threshold-like collapse in clearance; synaptic plasticity being unaffected by perturbations to YTHDF2 condensate properties; direct evidence that synaptic YTHDF2 condensation is nucleation-seeded rather than concentration-dependent, which would invalidate C-sat as the relevant pharmacological target even if the broader partitioning hypothesis survives; the predicted directional C-sat shifts—lowered in cognitive disorders, elevated in addiction—simply failing to appear; or evidence that YTHDF2-mediated decay proceeds through direct CCR4-NOT recruitment independent of condensate formation, which would invalidate the therapeutic premise.

## 4. Condensate Pharmacology: Feasibility Without Target Validation

Condensate-directed drug discovery is not science fiction anymore. It is a real, active field—and it has come a long way from the early speculation. In 2022, Dewpoint Therapeutics—a company built for exactly this—put out an industry perspective proposing condensate modulation as a new drug discovery approach [41]. Since then, the literature has started backing that up with real chemical matter.

Take TDP-43: small molecules that modulate its recruitment to stress granules prevent persistent TDP-43 accumulation in ALS/FTD cellular models [42]. Other small molecules dissolve stress granules via redox modulation and show benefit in ALS models [43]. Screening methods have kept pace: there are imaging-based, ML-assisted approaches for finding condensate-modulating compounds [44]. How do you drug an intrinsically disordered region without a binding pocket? It comes down to two principles. One: small molecules can shift a protein’s conformational ensemble. Two: condensates create distinct chemical environments. Compounds may partition into them based on a partition coefficient, though this is a design assumption rather than an established principle for biomolecular condensates. That changes the design problem for a YTHDF2-directed compound. You are engineering partitioning behavior, not hunting for a pocket. If we take that design principle seriously, it should shape the screening strategy. Partitioning would be measured as the ratio of compound concentration in the condensate phase to the concentration in the dilute phase—analogous to a logP but for a phase-separated compartment rather than an organic/aqueous boundary. Desirable compounds would show moderate partitioning (not extreme, which risks off-target accumulation) and would shift C-sat or nucleation kinetics at sub-stoichiometric concentrations. No such compound has been reported for YTHDF2, and it is unclear whether the partition-coefficient framework generalizes from organic solvents to biomolecular condensates without modification. We flag this as a design assumption, not an established approach. The reframing is conceptually compatible with the C-sat-centered logic proposed here. But let us be precise about what that compatibility means. It supports the general feasibility of the condensate-modulation approach as a chemistry category. It provides no target-specific support for YTHDF2, and, based on Section 3.1, it does not resolve whether bulk concentration is even the right parameter to modulate.

Three caveats limit how much weight this general feasibility evidence can carry.

First, every validated hit compound in this literature targets a protein with a disease-specific mutant or aggregation state. That provides a natural selectivity handle. YTHDF2 in the model proposed here has no such handle. The shared LCD composition across YTHDF1–3 (Section 2.1) makes within-family selectivity harder than current precedents address. We are not aware of any published condensate-modulating compound that targets a non-mutant, non-aggregating protein. If such a compound exists, it would substantially strengthen the feasibility argument; if it does not, the framework is asking the field to do something it has not yet done.

Second, none of the screening or hit-identification work surveyed here has been applied to YTHDF2 or FMRP. In any cell type.

Third, off-target condensate partitioning deserves explicit mention. If a small molecule partitions into YTHDF2 condensates, it will likely partition into other RNA-containing condensates with similar physicochemical properties—FMRP condensates, stress granules, P-bodies, processing bodies, and neuronal transport granules. The consequence is that the compound may perturb multiple condensate systems simultaneously, and the therapeutic effect observed in any experiment will reflect the combined perturbation. Without selectivity data across condensate types, it will be difficult to attribute any functional effect to YTHDF2 modulation specifically. Selectivity panels of the kind used in the ALS/FTD condensate-drug literature [41,42] provide a starting point, but they will need to be extended to cover the RNA-granule spectrum relevant to neurons.

One final point on blood–brain barrier penetration. LCD-binding chemotypes skew aromatic and planar, and this profile is generally associated with poor CNS penetration—though the quantitative relationship between chemotype and BBB permeability is complex and depends on multiple physicochemical parameters beyond aromaticity. We flag it as a design constraint, not as a disqualifying feature. If the screening campaign identifies compounds with favorable partition-coefficient profiles, BBB penetration will need to be assessed empirically, and the reference frame for CNS-targeted condensate modulators is still being established.

Table 2 compares the two strategies across five dimensions. The comparison clarifies what is established for catalytic inhibitors and what remains unproven for reader-condensate modulators.

## 5. A Staged Research Program

Given the objections raised in Section 2, Section 3 and Section 4, we are proposing a staged program (Figure 3). The goal: resolve the framework’s most fundamental uncertainties before committing to drug discovery targeting model 1. The program includes a parallel track (Tier 1b) for testing the network-mediated model, explicit sample size and replication requirements, and positive criteria alongside the failure criteria.

**Tier 1a—Does this actually happen in neurons? And if so, is it concentration-driven or nucleation-driven?**

We are talking super-resolution imaging of endogenous YTHDF2 and FMRP in cultured neurons: basal conditions; plasticity-stimulated conditions. The direct test: Spatially distinct condensates (model 1, Figure 2A)? Or co-localization (models 2–4, Figure 2B–D)?

Recent evidence has established that YTHDF2 is present in dendrites and at synapses [27,28,29], and that FMRP interacts with the YTHDF2 transport complex [29]. These findings provide the foundation for the imaging experiments. We will use antibody validation against YTHDF2 knockout neurons, plus a knock-in fluorescent tag if antibodies prove insufficient.

And in parallel: titration-based imaging—not just FRAP. Does YTHDF2 condensation follow classical concentration-threshold kinetics? Or are we looking at discrete nucleation events? If it is the latter, we need to rethink the pharmacological strategy in Section 4: shift toward nucleation-kinetics targets instead of C-sat.

**Limitation we must acknowledge.** Super-resolution imaging can reveal spatial segregation or co-localization, but it cannot distinguish co-occupancy (model 2) from kinetic competition (model 3). Both models predict co-localization; they differ in whether the two proteins bind the same transcript simultaneously or compete for binding sites at different rates. To distinguish them, we propose single-molecule imaging to measure binding and unbinding rates of YTHDF2 and FMRP on individual transcripts, complemented by biochemical assays (electrophoretic mobility shift assays, isothermal titration calorimetry) to measure competitive binding in vitro. These experiments are added as Tier 1b.

**Sample size and replication.** Our original power calculation treated neurons as independent while accounting for clustering with an intra-class correlation (ICC) of 0.4. With 30 neurons per condition across three cultures, the design effect is 4.6, giving an effective sample size of approximately 6.5 per condition. This is far below the approximately 64 per condition needed for 80% power at d = 0.5. We have therefore recalculated the sample size using independent cultures as the unit of analysis. Assuming ICC = 0.4 and 30 neurons per culture, at least 30 independent cultures per condition are required to achieve 80% power at α = 0.05 for d = 0.5. For d = 0.8, 11 independent cultures per condition are sufficient. We will use 30 independent cultures per condition for Tier 1a and Tier 1b, with 30 neurons per culture, and aggregate data at the culture level. For single-molecule imaging, we will analyze a minimum of 1000 individual transcript binding events per condition.

**Tier 1b—Test the network-mediated model in parallel, and distinguish model 2 from model 3**

The network-mediated model (model 4, Figure 2D) makes predictions that are different from those of model 1. Under model 4, FMRP loss should alter the expression or localization of multiple condensate-forming proteins, and transcript fate should shift as a downstream consequence of network perturbation rather than as a direct result of YTHDF2 competition. To test model 4, we will use proteomic and transcriptomic profiling of FMRP knockout neurons to identify network nodes whose expression or localization is altered. We will then perturb individual nodes using CRISPR interference (CRISPRi) or overexpression and measure transcript fate and YTHDF2 partitioning. If transcript fate shifts when network nodes are perturbed—even in the absence of direct YTHDF2/FMRP interaction—model 4 is supported. If transcript fate is unaffected by network perturbation but is affected by direct YTHDF2/FMRP competition, model 1 is supported. To distinguish model 2 (co-occupancy, Figure 2B) from model 3 (kinetic competition, Figure 2C), we will use single-molecule imaging to measure binding and unbinding rates of YTHDF2 and FMRP on individual transcripts. Under model 2, both proteins bind simultaneously, and the dissociation rates are similar. Under model 3, the two proteins compete for overlapping binding sites, and the dissociation rates differ. We will also use electrophoretic mobility shift assays and isothermal titration calorimetry (ITC) to measure competitive binding in vitro, under conditions that preserve condensate integrity.

**Sample size and replication.** For proteomic profiling, we will use three biological replicates per genotype. For network perturbation experiments, we will use 30 independent cultures per condition, with 30 neurons per culture, under the same power assumptions as those for Tier 1a. For single-molecule imaging, we will use 1000 binding events per condition as above.

**Tier 2—Quantify C-obs (if concentration-driven). Is it a regulated, disease-shiftable variable?**

Titration-based C-obs measurement in neurons at baseline. Then following plasticity-inducing stimuli. Parallel measurement in induced pluripotent stem cell (iPSC)-derived neurons carrying Rett-syndrome-associated methyl-CpG-binding protein 2 (MECP2) mutations against isogenic controls, and in nucleus accumbens neurons from chronic cocaine-treated mice versus saline controls (addiction model).

**How C-obs would be measured.** In the absence of a direct method for measuring dilute-phase concentration in neurons, we will use an indirect approach. YTHDF2 expression will be titrated across a range using an inducible promoter (Tet-ON) in cultured neurons, and condensate number and size will be quantified at each expression level. C-obs will be inferred as the expression level at which condensate number begins to increase sharply—the threshold below which YTHDF2 is diffuse and above which it condenses. This inference assumes that local concentration scales with total expression, which may not hold in a compartmentalized cell. To partially validate the inference, we will use FCS to estimate the local YTHDF2 concentration in the soma and in dendrites separately, and we will compare the inferred C-obs values across compartments. If the inferred values diverge substantially, the bulk-concentration assumption is challenged, and the framework pivots toward nucleation kinetics (see Section 3.1).

**Controls.** Non-condensate-forming YTHDF2 mutants (LCD-truncated), YTHDF1 and YTHDF3 paralogs, and a control protein with a similar LCD composition but no m6A-binding domain.

**Sample size and replication.** Six expression levels, 12 independent cultures per level, 25 neurons per culture. This provides 80% power for d = 0.8 under the same ICC assumptions.

**Tier 3—Screen for chemical matter**

Apply the imaging-based, ML-assisted screening approaches already validated for other condensate targets to the YTHDF2 LCD. Include counter-screens against FMRP, TDP-43, FUS, and hnRNPA1 LCDs. Use selectivity panels already established in the ALS/FTD condensate-drug literature. Include a partition-coefficient characterization for any hit compound and assess off-target partitioning against a panel of RNA-containing condensates (stress granules, P-bodies, and FMRP condensates).

**Sample size and replication.** Primary screen of 10,000–50,000 compounds in a 384-well format, with three replicates per plate and a Z′-factor ≥ 0.5 as the plate acceptance criterion. Hits will be confirmed in dose–response (eight concentrations, three replicates) and counter-screened against the selectivity panel.

**Tier 4—Functional and translational validation**

Begin with ex vivo electrophysiology in Rett-syndrome and Fragile X mouse hippocampal slices. Proceed to circuit-restricted genetic approaches in addiction models. But only after Tiers 1–3 return consistent positive signal.

**Sample size and replication.** For electrophysiology, a minimum of 12 slices from 6 animals per genotype per condition, based on standard power assumptions for field potential recordings (effect size d = 0.8, α = 0.05, β = 0.20).

**Positive criteria**

We have specified failure criteria in Section 3. Here we specify what would count as a positive result at each tier.

**Tier 1a positive result.** YTHDF2 and FMRP are spatially segregated in a majority of synaptic puncta under basal conditions, and co-localize transiently following plasticity stimulation.

**Tier 1b positive result (model 4).** FMRP loss alters the expression or localization of at least three condensate-forming network nodes, and perturbation of individual nodes shifts transcript fate in the predicted direction.

**Tier 2 positive result.** C-obs is measurable in neurons and shifts in the predicted direction (lowered in Rett-model neurons, elevated in addiction-model neurons). The inferred C-obs values are consistent across soma and dendrite, supporting the bulk-concentration assumption.

**Tier 3 positive result.** At least one compound shows dose-dependent modulation of YTHDF2 condensate formation with ≥10-fold selectivity over FMRP, TDP-43, FUS, and hnRNPA1, and with acceptable off-target partitioning.

**Tier 4 positive result.** Compound treatment rescues electrophysiological deficits in Rett-syndrome or Fragile X slices, in a manner dependent on YTHDF2 condensate modulation.

**Interpretation of ambiguous results**

If Tier 1a returns ambiguous results—YTHDF2 and FMRP sometimes co-localized and sometimes segregated, depending on the plasticity state or transcript—the interpretation depends on the pattern. If segregation correlates with a specific plasticity state, this supports context-dependent partitioning (a variant of model 1). If co-localization is the norm but with variable co-occupancy of individual transcripts, this favors model 2 or 3. If the pattern is random, this suggests the interaction is not the operative mechanism, and the framework should pivot to model 4 or be set aside.

We are trying to be honest about what this field can actually contribute in the near term. Tier 1 largely applies co-localization and reconstitution paradigms already used for other neuronal condensate pairs—FUS/FMRP interactions in ALS models being a direct precedent [26]—to a protein pair that has received less attention. That is legitimate. Fundable. Incremental methodology applied to a novel target pair. Not a conceptually new experimental approach. The field is better served by that framing. Better than an inflated claim of novelty.

## 6. Conclusions

What we have offered here is a hypothesis and a staged agenda: not a validated mechanism; not something ready for translational investment.

The biophysical foundations—YTHDF-family LLPS, m6A dependence, FMRP phase-separation competence, and the general druggability of condensate proteins—are in better shape now than when we first sketched this framework. But there are catches. The artificial conditions in those in vitro assays. The fact that it is family-wide, not YTHDF2-specific.

The synaptic claims are no longer untested. Recent evidence has established that YTHDF2 is present in dendrites and at synapses [27,28,29], that FMRP forms condensates in neurons, and that FMRP and YTHDF2 functionally interact in a competition-like manner [29]. The two prerequisites we previously identified as unestablished have been addressed by the field in the intervening period. What remains unresolved is the precise mechanism of competition—whether it operates through mutually exclusive partitioning, co-occupancy, or kinetic competition.

The biggest vulnerability remains: is bulk concentration really the right variable for synaptic YTHDF2 condensation? Or are we dealing with a nucleation-driven process that this framework does not touch? As far as we know, no one has raised this question in the literature. We also now recognize that YTHDF2-mediated decay may not require condensate formation at all, which would render the therapeutic premise moot. That possibility is listed as a failure criterion.

We see the uncertainties as reasons to prioritize experiments. Before more theory. Not reasons to give up. If the Section 3 failure criteria kick in—or if condensation is nucleation-driven, or if decay proceeds without condensation—we should set this framework aside. Go with whatever the data support.

But if not? Then condensate-directed modulation of m6A readers deserves a serious look. Early-stage, sure. But as a spatially specific complement to catalytic epitranscriptomic drugs. That is modest. Incremental. And it is all the evidence supports.

## Figures and Tables

**Figure 1:**
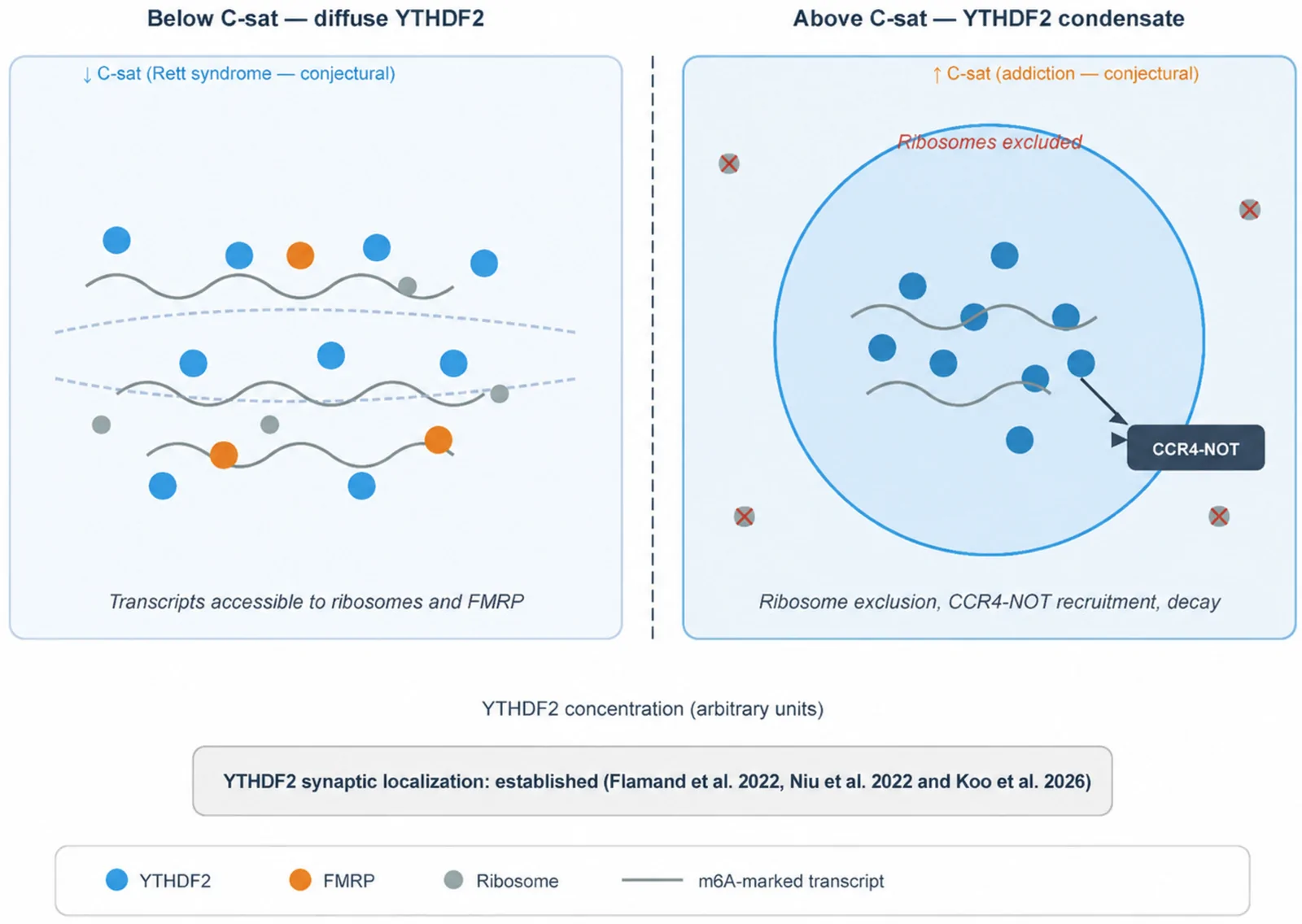
The Phase Transition Threshold model. Schematic of the proposed relationship between local YTHDF2 concentration, the saturation concentration (C-sat), and condensate formation at the synapse. Legend: YTHDF2 (blue circles), FMRP (orange circles), ribosomes (grey circles), and m6A-marked transcripts (grey wavy lines). Red “X” marks indicate ribosome exclusion. **Below C-sat (left panel):** YTHDF2 remains diffuse in the dendritic cytoplasm; m6A-marked transcripts are accessible to ribosomes and to FMRP, and are either translated or maintained in a translation-competent state. **Above C-sat (right panel):** YTHDF2 condensates (large blue circle) exclude ribosomes, recruit the CCR4-NOT deadenylase complex (black arrow), and promote transcript decay. While the figure depicts the two end states, the model posits an intermediate transition where local concentration approaches C-sat, prompting YTHDF2 to condense via its N-terminal low-complexity domain in an m6A-stimulated manner. Two competing scenarios for how cells cross the threshold are discussed in the text (Section 3.1)—a classical concentration-driven demixing (passive, well-mixed) and a nucleation-driven seeding event (scaffold-dependent, subcellularly localized)—but are not visually distinguished in this schematic. A pharmacological strategy targeting C-sat is only appropriate if concentration-driven condensation is confirmed. The blue (↓ C-sat) and orange (↑ C-sat) labels indicate the predicted direction of pathological C-sat shifts—lowered in cognitive disorders such as Rett syndrome, elevated in addiction models—both of which remain untested conjectures (Section 3.2). Support for the underlying biophysics: YTHDF2 is present in dendrites and at synapses (Flamand et al. 2022 [27]; Niu et al. 2022 [28]; Koo et al. 2026 [29]); FMRP forms condensates in neurons (Tsang et al. 2019 [25]); FMRP functionally antagonizes YTHDF2 in transcript fate (Koo et al. 2026 [29]).

**Figure 2:**
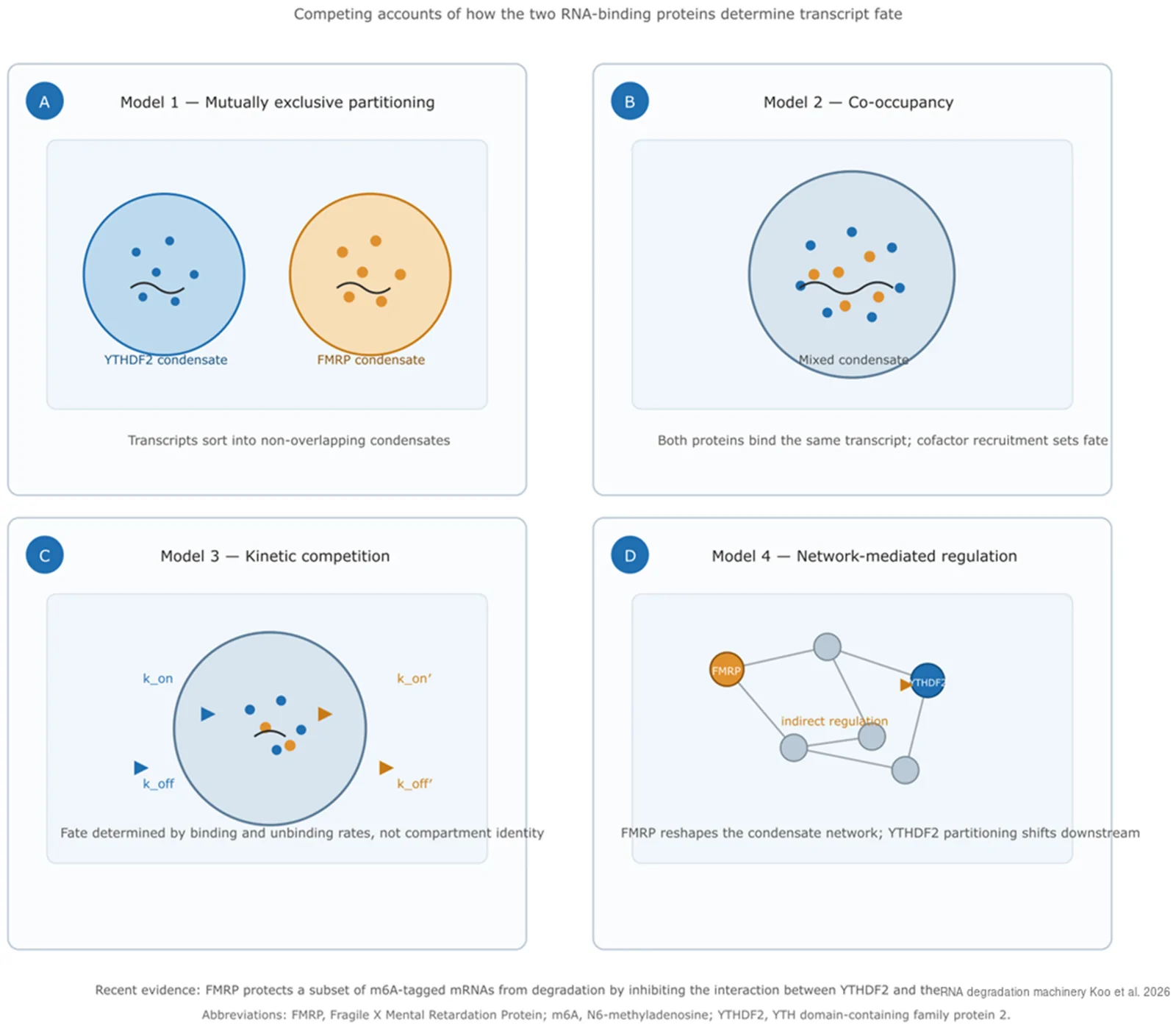
**Four models of YTHDF2/FMRP competition at the synapse.** Competing accounts of how the two RNA-binding proteins determine transcript fate. Legend: blue circles (YTHDF2), orange circles (FMRP), grey wavy lines (m6A-marked transcripts), and grey circles (other network proteins). Shaded circles represent condensates. **(A) Model 1—Mutually exclusive partitioning:** Transcripts sort into non-overlapping YTHDF2 (blue) or FMRP (orange) condensates. **(B) Model 2—Co-occupancy:** YTHDF2 and FMRP bind the same transcripts together within a mixed condensate, with downstream co-factor recruitment determining fate. **(C) Model 3—Kinetic competition:** Partitioning depends on binding and unbinding rates (represented by blue and orange triangles, labeled k_on and k_off), not on compartment identity. **(D) Model 4—Network-mediated regulation:** FMRP regulates multiple condensate-forming proteins (grey circles) that shape the synaptic RNA environment indirectly, with YTHDF2 partitioning shifting as a downstream consequence rather than through direct competition. Recent evidence from Koo et al. [29] demonstrates that FMRP protects m6A-tagged mRNAs from degradation by inhibiting the interaction between YTHDF2 and the RNA degradation machinery, providing direct support for a competition-like mechanism. This evidence is consistent with models 1, 2, and 3, and does not distinguish among them.

**Figure 3:**
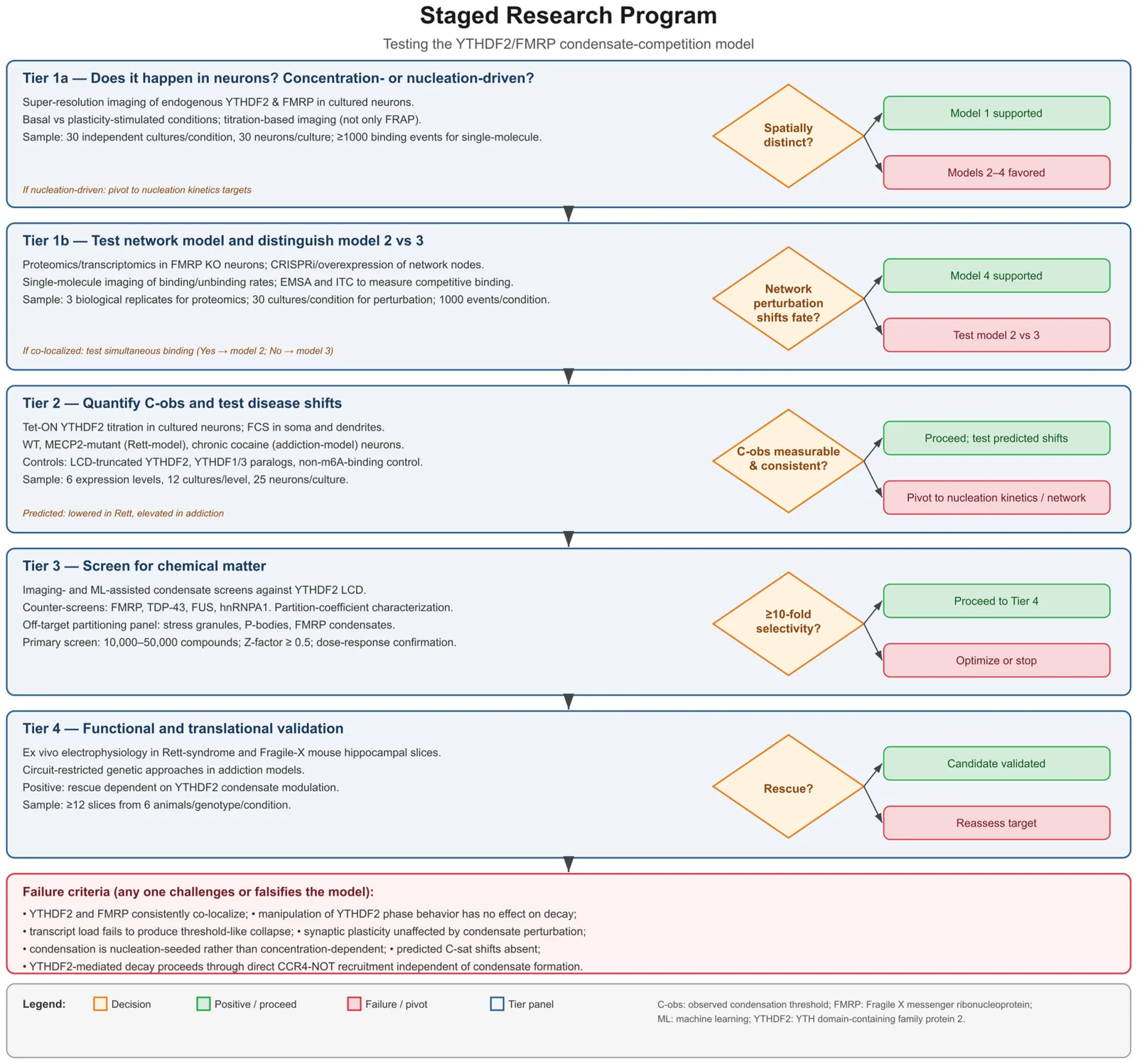
Staged research program for testing the YTHDF2/FMRP condensate-competition model. Tier 1a uses super-resolution imaging of endogenous YTHDF2 and FMRP in cultured neurons to test whether they form spatially distinct condensates (model 1) or co-localize (models 2–4) and whether YTHDF2 condensation is concentration-driven or nucleation-driven. Tier 1b tests the network-mediated model (model 4) and uses single-molecule imaging plus in vitro binding assays to distinguish co-occupancy (model 2) from kinetic competition (model 3). Tier 2 quantifies C-obs at baseline and after plasticity stimulation in wild-type, MECP2-mutant (Rett-model), and chronic-cocaine (addiction-model) neurons; failure to measure C-obs or compartment-divergent values triggers a pivot to nucleation kinetics. Tier 3 applies imaging- and ML-assisted condensate screens to the YTHDF2 low-complexity domain, with counter-screens against FMRP, TDP-43, FUS, and hnRNPA1 and off-target RNA-granule panels. Tier 4 validates function using ex vivo electrophysiology in Rett syndrome and Fragile X slices, followed by circuit-restricted genetic perturbation in addiction models. Decision points, positive criteria, and failure criteria are shown at each tier. Abbreviations: C-obs, observed condensation threshold; FMRP, Fragile X messenger ribonucleoprotein; ML, machine learning; YTHDF2, YTH domain-containing family protein 2.

**Table 1:** Tiered confidence assessment of the model’s constituent claims.

Claim	Evidence Tier	Supporting Observations	Key Limitation
YTHDF proteins undergo m6A-dependent LLPS in vitro and in cells	Strong—independently replicated across contexts, though under artificial conditions	LCD-driven droplet formation confirmed in vitro; m6A-enhanced condensation replicated across hepatic, arsenic-toxicology, and hematologic disease models [14,16,17,18,19]	Never directly demonstrated in neurons; in vitro conditions are non-physiological [20]; property is family-wide, not reader-specific; most cellular evidence comes from overexpression systems, though recent work has begun to address this [21]
FMRP undergoes phase separation and represses translation via a condensed state	Moderate—recent evidence demonstrates condensate formation in neurons	In vitro droplet formation from purified FMRP LCD [25]; established, non-condensate translational-repression mechanism independently confirmed by CLIP-seq [23]; FMRP forms ribosome-rich condensates in axons and dendrites	Causal necessity of the condensed state, versus correlation with it, has not been established [25,26]
YTHDF2 is present in dendrites and at synapses	Strong—multiple independent studies	Flamand & Meyer [27] identified YTHDF2 as mediator of neuronal mRNA localization; Niu et al. [28] showed YTHDF2 expression in retinal ganglion cells and effects on dendrite development; Koo et al. [29] demonstrated YTHDF2 in neuronal transport complexes	The precise synaptic localization and dynamics remain to be fully characterized
m6A dysregulation contributes to synaptic plasticity deficits and cognitive disease	Strong	Multiple independent lines link m6A loss to reduced synaptic-transcript translation, impaired plasticity, and psychiatric/neurodegenerative disease association [31,32,33,34]	Mechanism is consistently attributed to writer/eraser stoichiometry, not reader partitioning; Alzheimer’s evidence is correlational, and direction of causality is unresolved
YTHDF2 and FMRP compete for m6A-marked transcripts	Moderate—recent evidence supports functional antagonism	Koo et al. [29] reported that FMRP reshapes the YTHDF2 complex by recruiting cofactors and motor proteins, promoting transport over degradation	Whether competition operates through mutually exclusive partitioning (model 1), co-occupancy (model 2), or kinetic competition (model 3) remains unresolved
Bulk concentration (C-sat), rather than nucleation kinetics, is the operative variable governing synaptic YTHDF2 condensation	Unestablished	Assumed by the framework; not tested	No data exist distinguishing nucleation-driven from concentration-driven condensation for YTHDF2 in any cell type; true C-sat measurement in neurons is not currently feasible; C-obs is an indirect proxy

Abbreviations: C-sat, saturation concentration; FMRP, Fragile X Mental Retardation Protein; LCD, low-complexity domain; LLPS, liquid–liquid phase separation; m6A, N6-methyladenosine; YTHDF, YTH domain-containing family protein.

**Table 2:** Catalytic versus reader-condensate targeting strategies, compared on equivalent terms.

Dimension	Catalytic (METTL3/FTO) Inhibitors	Reader-Condensate (YTHDF2 LLPS) Modulators
Clinical stage	Phase 1, oncology (STC-15) [2,3]	Preclinical; target validation not initiated
Mechanism of specificity	Structural (defined catalytic pocket)	Biophysical (partition coefficient/C-sat shift); plausible in principle, target-unproven, and dependent on the unresolved nucleation-versus-concentration question
Demonstrated selectivity	Lineage-selective downstream effects reported in hematopoiesis [5]	Not demonstrated in any tissue; within-family (YTHDF1/2/3) selectivity is an unresolved chemistry problem inherent to the shared LCD mechanism
Screening/discovery infrastructure	Mature SAR-based pipeline	Emerging imaging- and ML-assisted condensate screens exist for other IDR targets [39,40,43] but have not been applied to this target; no published example of a condensate modulator against a non-mutant, non-aggregating target
CNS delivery precedent	Oral small molecule (STC-15)	Untested; LCD-binding chemotypes skew aromatic/planar—a profile associated with poor blood–brain barrier penetration

Abbreviations: CNS, central nervous system; FTO, fat mass and obesity-associated protein; LLPS, liquid–liquid phase separation; METTL3, methyltransferase-like 3; SAR, structure–activity relationship; YTHDF2, YTH domain-containing family protein 2.

## Data Availability

The original contributions presented in this study are included in the article. Further inquiries can be directed to the corresponding author.

## Abbreviations

**Abbreviation**	**Full term**
C-sat	Saturation concentration
CNS	Central nervous system
CRISPRi	CRISPR interference
FCS	Fluorescence correlation spectroscopy
FMRP	Fragile X Mental Retardation Protein
FRAP	Fluorescence recovery after photobleaching
FTO	Fat mass and obesity-associated protein
IDR	Intrinsically disordered region
iPSC	Induced pluripotent stem cell
ITC	Isothermal titration calorimetry
LCD	Low-complexity domain
LLPS	Liquid–liquid phase separation
m6A	N6-methyladenosine
MECP2	Methyl-CpG-binding protein 2
METTL	Methyltransferase-like protein
PEG	Polyethylene glycol
PTEN	Phosphatase and tensin homolog
SAR	Structure–activity relationship
YTHDF	YTH domain-containing family protein
